# Wenfei Buqi Tongluo Formula Against Bleomycin-Induced Pulmonary Fibrosis by Inhibiting TGF-β/Smad3 Pathway

**DOI:** 10.3389/fphar.2021.762998

**Published:** 2022-01-21

**Authors:** Lu Ding, Yaxin Li, Yingying Yang, Siyu Song, Hongyu Qi, Jing Wang, Ziyuan Wang, Jiachao Zhao, Wei Zhang, Linhua Zhao, Daqing Zhao, Xiangyan Li, Zeyu Wang

**Affiliations:** ^1^ Jilin Ginseng Academy, Key Laboratory of Active Substances and Biological Mechanisms of Ginseng Efficacy, Ministry of Education, Jilin Provincial Key Laboratory of Bio-Macromolecules of Chinese Medicine, Changchun University of Chinese Medicine, Changchun, China; ^2^ College of Integrated Traditional Chinese and Western Medicine, Changchun University of Chinese Medicine, Changchun, China; ^3^ Graduate College, Beijing University of Chinese Medicine, Beijing, China; ^4^ Department of Respiratory, Affiliated Hospital of Changchun University of Chinese Medicine, Changchun, China; ^5^ College of Traditional Chinese Medicine, Changchun University of Chinese Medicine, Changchun, China; ^6^ Department of Scientific Research, Changchun University of Chinese Medicine, Changchun, China; ^7^ Molecular Biology Laboratory, Guang’anmen Hospital, China Academy of Chinese Medical Sciences, Beijing, China

**Keywords:** WBT formula, network pharmacology, pulmonary fibrosis, extracellular matrix accumulation, epithelial–mesenchymal transition, TGF-β1/Smad3 pathway

## Abstract

Pulmonary fibrosis (PF) is the end stage of various chronic and progressive interstitial lung diseases. TGF-β, a profibrotic cytokine, can promote epithelial–mesenchymal transition (EMT), extracellular matrix (ECM) accumulation, and fibroblast proliferation, which contribute to progressive lung remodeling in PF. The Wenfei Buqi Tongluo (WBT) formula has been certified to be effective in the prevention and treatment of PF in clinical practice and has inhibitory effects on EMT, inflammation, and profibrotic factors. However, the pharmacological mechanisms of WBT against PF need to be further explored. In this study, we first analyzed the chemical components of the WBT formula using the UHPLC/Q-TOF-MS analysis. The potential targets of the identified compounds from WBT were predicted by the network pharmacology, which was confirmed by *in vivo* and *in vitro* study. After screening by the PubChem database, we first identified the 36 compounds of WBT and predicted the TGF-β signaling pathway, with ECM degradation as potential mechanism of WBT against PF by the network pharmacology. Furthermore, WBT treatment inhibited the levels of TGF-β and Smad3 phosphorylation and subsequently alleviated EMT and ECM accumulation in the bleomycin-induced mouse model and TGF-β1–induced cell model. These findings indicate that WBT can block the progressive process of PF by inhibiting EMT and promoting ECM degradation *via* the TGF-β/Smad3 pathway. This study may provide new insights into the molecular mechanism of WBT for the prevention and treatment of PF in the clinical application.

## Introduction

Pulmonary fibrosis (PF) is an end stage for various chronic and progressive interstitial lung diseases and is accompanied by gradually worsening symptoms such as fatigue, weight loss, and shortness of breath (King et al., 2011). PF is caused by occupational factors, autoimmune disorders, infections, and genetic factors, but the cause of this disease generally cannot be identified, which is called idiopathic PF (IPF) (Glass et al., 2020). IPF is a specific and common type of PF, in which lung function inexorably declines, leading to respiratory failure and eventually death. The incidence of IPF is increasing every year, with approximately three million people worldwide suffering from the disease (Nalysnyk et al., 2012; Hutchinson et al., 2015). Currently, only two drugs, pirfenidone and nintedanib, are used for the treatment of IPF, which can slow the decline of lung function in patients with IPF (King et al., 2014; Flaherty et al., 2019). However, the effects of the two drugs on patient survival are uncertain and can produce many adverse reactions, such as nausea, diarrhea, dyspepsia, and rash (Pang et al., 2019). Therefore, it is essential to develop drugs to delay the progression of IPF.

As reported, epithelial–mesenchymal transition (EMT), myofibroblast activation, and collagen accumulation are main pathological characteristics of PF (Ohyashiki et al., 1976; Todd et al., 2012; Salton et al., 2019). During the development of PF, EMT is a pathological process where epithelial cells lose their phenotypes and acquire mesenchymal features. EMT is often detected for several biomarkers, including E-cadherin, N-cadherin, and α-smooth muscle actin (α-SMA) (Salton et al., 2019). An aberrant EMT event can induce extracellular matrix (ECM) accumulation and deposition, which contributes to the progression of lung remodeling in patients with PF (Philp et al., 2018). Myofibroblasts, differentiated from fibroblasts, are the main producers of ECM and are characterized by the presence of α-SMA (Todd et al., 2012). Importantly, TGF-β, as a profibrotic cytokine, can promote EMT, ECM accumulation, and fibroblast proliferation and differentiation to myofibroblasts to participate in PF, which may be mediated by the Smad2/3 signaling pathway (Kolahian et al., 2016; Walton et al., 2017; Lederer and Martinez, 2018).

Traditional Chinese medicine (TCM) has a long history in treating PF and its practitioners have accumulated rich experience in doing so. TCM considers the fact that the pathogenesis of PF is the obstruction of *Qi* and the stagnation of blood circulation, and the treatment for PF needs to promote *Qi* and activate blood circulation (Zhang et al., 2018). Moreover, many TCM drugs, including formulas (Fufang Biejia Ruangan Pills, Jinkui Shenqi Wan, etc.), single herbs (*Rheum palmatum* L., *Astragalus mongholicus* Bunge, etc.), and active components (gallic acid, quercetin, curcumin, gambogic acid, etc.) have been identified to be efficient in anti-fibrosis, improving lung function and reducing dyspnea (Zhang et al., 2021). The Wenfei Buqi Tongluo (WBT) formula is a hospital preparation for PF made by the Affiliated Hospital of Changchun University of Chinese Medicine that has been certified to be effective in anti-fibrosis treatment in clinical practice. As shown in Table 1, WBT is composed of 13 Chinese medicines. Our previous study showed that WBT could inhibit EMT in TGF-β1–induced A549 cells (Ding et al., 2021). In addition, the Xian-ke granule, a basic TCM formula of WBT, proved to be effective in anti-PF for bleomycin (BLM)–induced mice through the inhibition of the inflammatory response (Li et al., 2012) and the decrease of the connective tissue growth factor and integrin-linked kinase in the lung tissues (Yang et al., 2014). These results indicate that WBT has potential anti-fibrosis effect, but its pharmacological mechanisms and possible components need to be further explored.

**TABLE 1:** The compositions of WBT formula.

Chinese name	Abbr	Latin name	Family	Weight (g)	Part used	Voucher specimen
Huang qi	HQ	*Astragalus mongholicus* Bunge	Fabaceae	40	Root	201910-01
Huang qin	HQN	*Scutellaria baicalensis* Georgi	Lamiaceae	20	Root	201910-02
Dan shen	DS	*Salvia miltiorrhiza* Bunge	Lamiaceae	20	Root	201910-03
Hu zhang	HZ	*Reynoutria japonica* Houtt.	Polygonaceae	15	Rhizome	201910-04
Dang gui	DG	*Angelica sinensis* (Oliv.) Diels	Apiaceae	15	Root	201910-05
Chuan xiong	CX	*Conioselinum anthriscoides ‘Chuanxiong’*	Apiaceae	15	Root	201910-06
Di long	DL	*Pheretima aspergillum* (E.Perrier)	Megascolecidae	10	Whole animal	201910-07
Tao ren	TR	*Prunus persica* (L.) Batsch	Rosaceae	10	Seed	201910-08
Zi wan	ZW	*Aster tataricus* L.f.	Asteraceae	15	Rhizome	201910-09
Kuan donghua	KDH	*Tussilago farfara* L.	Asteraceae	15	Flower	201910-10
Jiang banxia	JBX	*Pinellia ternata* (Thunb.) Makino	Araceae	9	Rhizome	201910-11
Wei lingxian	WLX	*Clematis chinensis* Osbeck	Ranunculaceae	15	Rhizome	201910-12
Xi xiancao	XXC	*Sigesbeckia orientalis* L.	Asteraceae	15	Herba	20191013

The network pharmacology is a meaningful way to reveal the pharmacological mechanism of TCM formula, and the holistic view is a common characteristic between the TCM and the network pharmacology (Li and Zhang, 2013). Therefore, in this study, we used UHPLC/Q-TOF-MS to identify the possible components in WBT. The pharmacology network was used to predict the potential targets and mechanisms of WBT against PF, according to the guidance of the network pharmacology evaluation method (Li, 2021). Then, the pharmacological function and possible mechanism of WBT against PF was confirmed in animal and cell experiments. This study may provide new insights into the therapeutic effect and molecular mechanisms of WBT in the clinical applications aiming to delay PF progression.

## Materials and Methods

### Materials and Reagents

Collagen I (ab34710), α-SMA (ab5694), E-cadherin (ab40772), N-cadherin (ab76011), TGF-β1 (ab215715), p-Smad3 (S423/S425, ab52903), and goat anti-rabbit/mouse antibodies (ab6721, ab6789) were purchased from Abcam (Cambridge, MA, USA). BLM (HY17565) and pirfenidone (PFD, HYB0673) were obtained from MedChemExpress (Princeton, NJ, USA). Calycosin-7-glucoside (ST08820120-5240), scutellarin (RS03111020-5839), apigenin-7-O-β-D-glucuronic acid (ST08920120-3075), oroxin B (ST09240120-5589), scutellarin methylester (ST15580120-4087), baicalin (RS01861020-5587), salvianolic acid A (ST005570120MG-4881), emodin glucoside (ST10890120-7823), wogonoside (ST08350120-5014), apigenin (RS000411020-5729), scutellarein (ST06340120-3414), baicalein (RS01871020-5699), isoastragaloside IV (ST81100105-6855), wogonin (RS01711020-5325), oroxylin A (ST23660220-6180), emodin (RS01401020-5940), salvianolic acid F (ST22410105mg-5189), salvianolic acid B (ST000500120mg-4065), and luteolin (RS00071020-5864) were purchased from Shanghai Shidande Standard Technical Service Co., Ltd. (Shanghai, China). Astragaloside I (DST190216-016), astragaloside II (DST180315-023), and astragaloside III (DST181118-018) were purchased from Chengdu Durst Biotechnology Co., Ltd. (Chengdu, China).

### Preparation of WBT Extract

The 13 Chinese medicines (Table 1) constituting WBT were purchased from Beijing General Pharmaceutical Corporation (Beijing, China) and provided by the Department of Pharmacy, the Affiliated Hospital of Changchun University of Chinese Medicine (Changchun, China). The voucher specimens were deposited at the Jilin Ginseng Academy, Changchun University of Chinese Medicine. According to the preparation processing as traditional use, the standard procedure from Chinese Pharmacopoeia (2020 edition), and previous studies (Huang Q. et al., 2018; Yang et al., 2021), all drugs were blended, mixed, and extracted by 2,000 ml of distilled water (the drug solvent ration of this formula is 9.0) at 100°C for 30 min, which was repeated three times to obtain the aqueous extract of WBT formula. After centrifugation, the supernatant was dried under a vacuum to produce a powdery extract with the drug-extract ratio of 22.47% (obtained 48 g of powdery extract from 214 g of raw materials). The powder was stored at −20°C for further experiments.

### UHPLC/Q-TOF-MS Analysis of WBT Extract

As previous described (Zuo et al., 2019; Yang et al., 2021), UHPLC analyses were carried out with a Waters ACQUITY UPLC I-Class Plus/Xevo G2-XS QTOF system (Waters, Milford, USA). Chromatographic separation was performed on a Waters ACQUITY UPLC BEH C18 (4.6 × 150 mm, 3.5 μm) at 30°C. The mobile phase was composed of 0.1% formic acid in water (A) and 0.1% formic acid in acetonitrile (B) running at 0.3 ml/min, consistent with the following optimal gradient elution program: 0–4 min, 1% B; 4–5 min, 1%–5 B; 5–10 min, 5%–17 B; 10–18 min, 17%–17 B; 18–23 min, 17%–22 B; 23–28 min, 22%–25 B; 28–34 min, 25%–32 B; 34–37 min, 32%–50 B; 37–40 min, 50%–95 B. The injection volume of WBT samples was 5 µl. The high-resolution MS data were recorded on a Xevo G2-XS QTOF mass spectrometer by MS^E^ in both the positive and negative ESI modes. The parameters of the electrospray ion source were set as follows: capillary voltage, 2.5 kV; cone voltage, 60 V; collision energy, 40–80 eV; scan mass range, 100–1,500 m/z; ion source temperature, 120°C; desolvation gas temperature, 500°C; cone gas flow rate, 50 L/h; desolvation gas flow rate, 800 L/h. The obtained MS^E^ data were conducted using UNIFI™ 1.9.3.0 software (Waters, Milford, USA).

### Components and Disease Targets Collection

The PubChem database (https://pubchem.ncbi.nlm.nih.gov/) was used to obtain the molecular structure (SDF format) of each component that identified from WBT powder extract. Then, the molecular structural files were uploaded to the PharmaMapper (http://lilab-ecust.cn/pharmmapper/submitfile.html), ChemMapper (http://lilab-ecust.cn/chemmapper/), and SwissTarget (http://www.swisstargetprediction.ch/index.php) databases to predict the potential targets of each component from WBT (Huang H. et al., 2018; Yang et al., 2020; Mu et al., 2021). The GeneCards database (https://www.genecards.org/) was used to collect PF-related targets (Mu et al., 2021). Finally, the corresponding Gene and UniProt ID of each target were obtained from the UniProt database (https://www.uniprot.org/) (Shawky, 2019). The targets were used for the establishment of a component-target network.

### Network Construction and Analysis

The targets of WBT and PF were combined so that the overlapping targets between PF and WBT could be obtained. Then, the overlapping targets were uploaded to the STRING database, and the information about protein–protein interaction was obtained. Meanwhile, the network of Chinese medicines, chemical ingredients, and potential targets was established and visualized by Cytoscape 3.8.0. The components of WBT and targets of the components were represented by hexagon and (or) rectangle, and the interaction was shown by a connecting line. Finally, depending on the overlapping targets, the significant Gene Ontology (GO) and Reactome Pathway were screened by the Metascape and DAVID databases.

### Mouse Model Establishment and Drug Administration

All animal care and procedures were approved by the Experimental Animal Ethics Committee of Changchun University of Chinese Medicine (batch number: 20190134). Seventy of 8-week-old male C57BL/6 mice purchased from Beijing Weitong Lihua Experimental Animal Technology Co., Ltd. [License No. SCXK (Beijing) 2016-0006] were kept at the Animal Experimental Center of Changchun University of Chinese Medicine (Changchun, China) at an ambient temperature (20 ± 2°C) and 50%–60% humidity. In this study, mice were randomly divided into seven groups with 10 mice in each group, including the blank control, sham operation, BLM, BLM + WBT (3, 6, and 12 g/kg, among them, the dose of 6 g/kg/day is equal dosage of clinic, 12 g/kg/day is the higher dosage, and 3 g/kg/day is the lower dosage), and BLM + pirfenidone (PFD, 200 mg/kg) groups, according to the book named Experimental Methodolgy of Pharamacology (2002 version). Except for the control group, mice were intraperitoneally with pentobarbital sodium (10 ml/kg) and intratracheally injected with BLM (5 mg/kg) or 0.9% normal saline (sham operation group), according to the previous report (Shahbaz et al., 2021). WBT and PFD were administered by gavage on the second day after BLM or NS injection for 14 days (once a day). Mice in the blank control, sham, and model groups were given pure water in the same amount of WBT.

### Micro-CT Imaging

Micro-CT imaging was performed with Quantum FX Micro CT (PerkinElmer, Inc., Waltham, MA, USA). After anesthetizing with isoflurane, the lungs were imaged with the help of cardiopulmonary gating techniques. The X-ray system of the scanner used a micro-focusing tube with a focal spot of 5 μm at 4 W and generated X-rays with cone beam geometry. The scanner’s detection system was composed of an amorphous silicon digital X-ray plate, which could acquire projection radiographs at a rate of 30 frames per second. The X-ray tube was set at 90 kV and 80 μA and took the projection radiographs during a 360° gantry rotation, with a total scanning time of 4 min. The reconstructed visual field was 36 mm with a high-resolution scanning mode (Lv et al., 2013; Ruscitti et al., 2017).

### Histopathological Examination and Assessment

H&E and Masson’s trichrome stainings were performed to evaluate the pathological changes of mouse lung tissues from different groups, as previously described (Zhao et al., 2020). For H&E and Masson’s trichrome staining, the exfoliated lung tissue of mice was fixed in 4% paraformaldehyde for 24 h, embedded in paraffin, cut into the 5-µm-thick sections, and stained with H&E and Masson’s trichrome kits. The degrees of inflammatory and fibrotic injuries in the lung tissues were scored according to the Mikawar and Ashcroft methods, respectively (Katoh et al., 1998; Pedroza et al., 2016). Immunohistochemical (IHC) staining was used to analyze the levels of PF-related proteins, such as collagen I, α-SMA, E-cadherin, N-cadherin, and TGF-β1 in the lung tissues, according to the manufacturer’s instructions (Kishi et al., 2018). After dewaxing and antigen repairing, the tissue was incubated with primary antibodies at 4°C overnight, followed by a conjugated secondary antibody and DAB staining. After counterstaining with hematoxylin, the images for antigenic sites were visualized and acquired by a Digital Microscope and Slide Scanner (M8, PreciPoint, Thuringia, Germany). The relative staining score of each protein was determined by calculating the areal density using Image-Pro Plus 6.0 software (Media Cybemetics, Inc., Rockville, MD, USA).

### Hydroxyproline Analysis

The content of hydroxyproline (HYP) in mouse lung tissue was quantified by the HYP assay kit (Nanjing Jiancheng Institute of Bioengineering), according to a previous study (Lv et al., 2013).

### Cell Culture and Model Establishment

The TC-1 cell line was bought from the iCell Bioscience, Inc. (Shanghai, China), and cultured in RPMI 1640 containing 10% fetal bovine serum (Clark Bioscience, Claymont, USA), penicillin (100 kU/L), and streptomycin (100 mg/L) (Biosharp, Hefei, China) at 37°C in a 5% CO_2_ humidified incubator. When the cells were in good condition, in accordance with the previous study (Zeng et al., 2021), TGF-β1 (10 ng/ml) was added to induce TC-1 cells transformation into mesenchymal-like cells.

### Cell Viability Assay

According to the previous study (Zeng et al., 2021), TC-1 cells were seeded into 96-well plates at a density of 6,000 cells/well, which were used to investigate the effect of WBT on cell viability after treatment for 48 h. To investigate the toxicity of WBT, TC-1 cells were treated with WBT at a concentration of 7.1–1,000 μg/ml for 48 h. In the TGF-β1–induced cell model, TC-1 cells were set as the control [dimethyl sulfoxide (DMSO) alone], model [TGF-β1 (10 ng/ml)], and treatment group [TGF-β1 (10 ng/ml) + WBT (7.1–1,000 μg/ml)] to examine the effect of WBT on the cell viability. After treatment for 48 h, MTT (0.5 mg/ml) was added (Solibol, Beijing, China), and DMSO (150 μl) was used to dissolve formazan crystals, which was measured as the absorbance at 490 nm using a microplate reader. The cell survival rate was calculated as the percentage of each group relative to the control group.

### Quantitative Real-Time PCR Analysis

The total RNA of TC-1 cells in different groups was extracted by a total RNA kit. After that, 1 μg of total RNA was reverse-transcribed into cDNA with the iScript cDNA synthesis kit. The Bio-Rad CFX96 system was used to perform quantitative (qPCR) analysis, and the relative mRNA level was calculated using the 2^−ΔΔCt^ method and normalized by GAPDH (Zhang Z. et al., 2020). The primer sequences of N-Cadherin, E-cadherin, α-SMA, collagen I, and GAPDH are listed in Supplementary Table S1.

### Western Blot Analysis

The total protein of TC-1 cells in different groups was obtained by an ice-cold RIPA buffer and separated by SDS-PAGE, transferred onto PVDF membranes. After blocking for 1 h at room temperature with a blocking buffer containing 5% BSA, the membranes were incubated with the primary antibody overnight at 4°C. After incubating with the HRP-conjugated secondary antibody for 2 h, the blots were visualized by a chemiluminescent imaging system (ChemiDoc XRS+, Bio-Rad, CA, USA) and quantified by ImageJ software (Jiang et al., 2021).

### Statistical Analysis

The data are expressed as mean ± SD. All data were statistically analyzed by GraphPad prism 9.0 software (San Diego, CA, USA). Multiple groups were compared by one-way ANOVA (Tukey’s post hoc) to determine the statistical significance. For all the statistical analyses, *p* < 0.05 was statistically significant.

## Results

### Chemical Components Identification and Similarity Analysis of WBT

**FIGURE 1:**
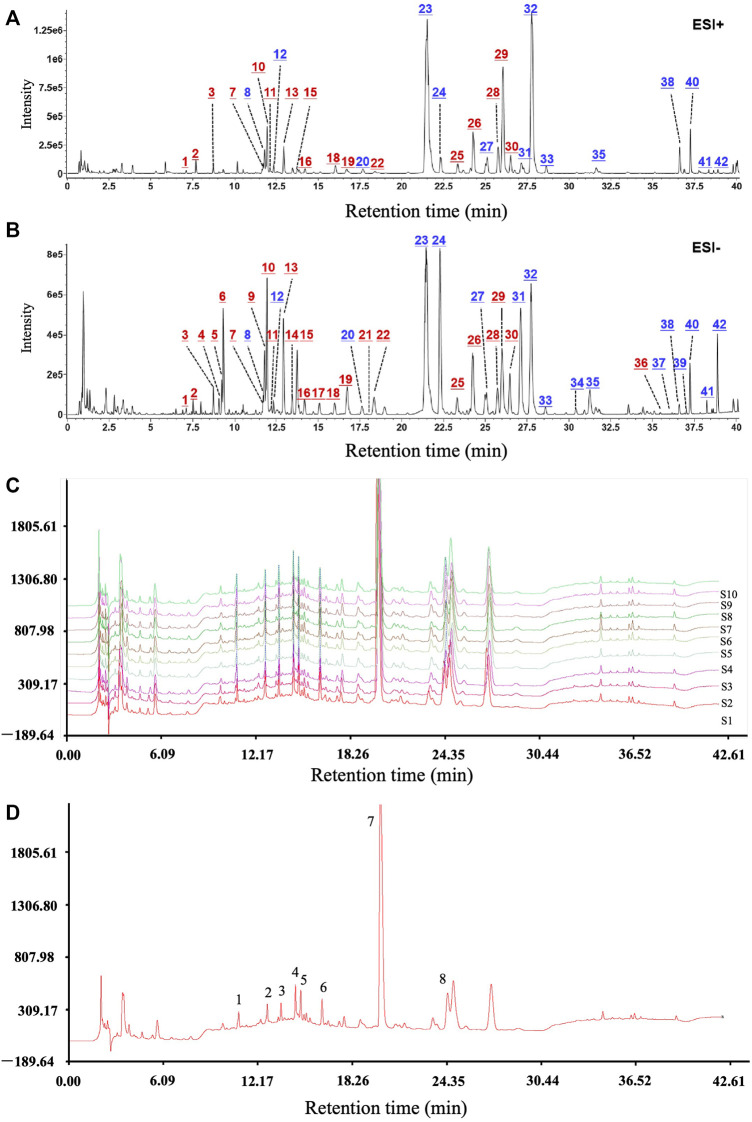
The main active components and good reproducibility of the WBT formula were determined by UHPLC/Q-TOF-MS analysis. **(A,B)** The chromatograms of the WBT formula for 42 active components in positive and negative ion modes. Blue numbers represent the compounds confirmed with the reference standards. **(C and D)** The reproducible HPLC fingerprints of the 10 batches of WBT (S1–S10) were identified using the Similarity Evaluation System for Chromatographic Fingerprint of Traditional Chinese Medicine (2004 Edition).

After optimization of the chromatographic and mass spectrometric conditions, a total of 42 compounds were identified or deduced from WBT formula, in which 16 components (expressed by * in Supplementary Table S2), including calycosin-7-glucoside, scutellarin/methylester, baicalin, and salvianolic acid A/B, were identified by the reference standards. The 42 compounds in WBT were characterized by comparing the retention time, accurate mass, and fragment ions with those of the standards or data reported by the literature. Furthermore, the attribution to the single herb was confirmed by the base peak intensity (BPI) chromatograms of the blank sample, WBT formula, and individual herbs. The BPI chromatograms of the WBT formula after collection in the positive and negative ion modes are shown in Figures 1A,B. The detailed information for 42 compounds in WBT is shown in Supplementary Table S2. In addition, the fingerprint and the similarity of the WBT formula were further analyzed by UHPLC method. As shown in Figures 1C,D, the percentages of the similarities from all 10 batches of WBT powdery extract were range from 91.0% to 99.3%, which indicates that the WBT powdery extract has good reproducibility. Importantly, we selected six standard compounds in WBT extract to perform quantitative analysis to avoid the risk of artifacts with higher dose in animal study. As shown in Supplementary Figure S1 and Supplementary Table S3, the WBT extract included calycosin-7-glucoside (0.4499 mg/g), baicalin (17.2408 mg/g), salvianolic acid B (3.3389 mg/g), salvianolic acid A (0.96 mg/g), emodin-8-β-D-glucoside (1.0735 mg/g), and wogonoside (6.7344 mg/g), which were the dose range as indicated in the consensus document of clinical evidence-based ethnopharmacology.

### Potential Target Screening and Network Establishment for Components of WBT Formula and Pulmonary Fibrosis

**FIGURE 2:**
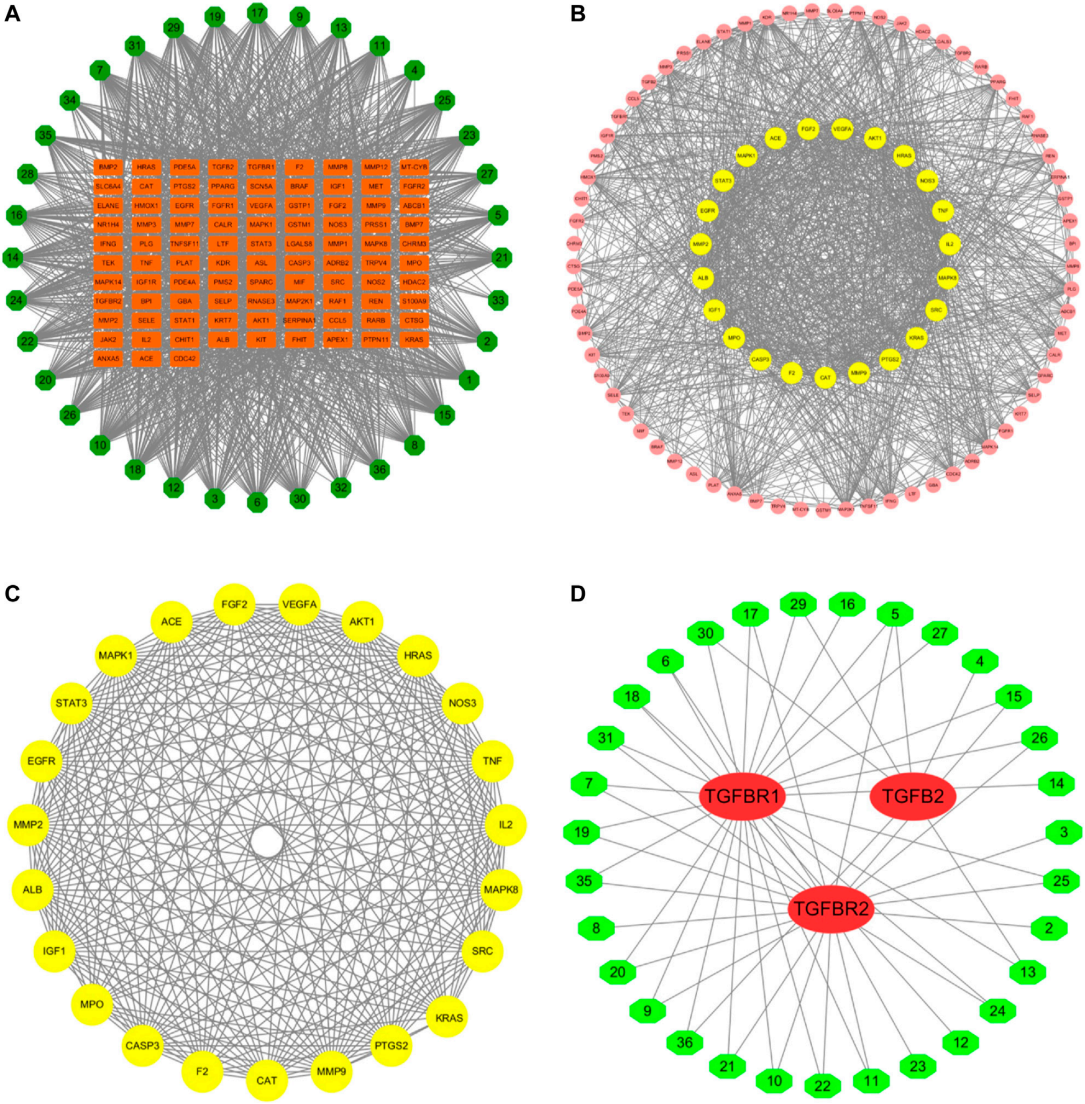
Potential targets of WBT formula were predicted by network pharmacology. **(A)** The network of the 36 selected components of WBT formula and potential targets was constructed. The green octagons represent the components ID of 36 compounds, and the orange rectangles represent the potential targets participated in PF. **(B and C)** PPI networks of 93 potential targets and 23 core targets are shown after screening with the degree ≥27, betweenness ≥0.08, and closeness ≥0.58. The circle represents the all-potential targets, and the yellow circle represents the core targets **(D)**. The network of 31 active components and the targets in TGF-β pathway was analyzed. The red ovals represent the TGF-β–related targets, and the green hexagons represent the 31 screened components from WBT formula.

According to the PubChem database, the 42 components in the WBT powdery extract were normalized, and those that had not been confirmed potential therapeutic targets were excluded, leaving 36 ingredients for further analysis of the network pharmacology (Table 2). The molecular structure files of the 36 components were obtained from the PubChem database, depending on which the potential targets of 36 components were obtained from several databases, such as PharmaMapper database, ChemMapper database, and SwissTarget database. Moreover, the potential targets were screened according to many parameters (fit score ≥2.5 in the PharmaMapper database, score ≥0.01 in the ChemMapper database, probability ≥0.079 in the SwissTarget database). Afterward, the UniProt ID corresponding to each target was obtained from the UniProt database, and the reduplicative targets were deleted to obtain a total of 687 potential therapeutic targets of 36 components of the WBT powdery extract (Supplementary Table S4). Subsequently, the network of 36 components and their 687 potential targets was constructed (Supplementary Figure S2). In addition, according to the score >11.8, a total of 584 potential targets of PF were selected from the GeneCards database. On the basis of the 687 potential targets of WBT and the 584 potential targets of PF, 93 overlapping targets were obtained, and the network of 36 active components and 93 overlapping targets was established and is shown in Figure 2A and Table 3. Importantly, on the basis of the topological analysis for component-overlapping targets network, the top four components of WBT formula were screened out depending on the degree >48, including baicalein, oroxylin A 7-O-β-D-glucuronide, viscidulin I, and viscidulin III (Supplementary Figure S3). As shown in Figure 2B, the PPI network of potential targets from WBT formula has 93 nodes and 1,272 edges. The 23 main targets, represented by yellow circles, contained ALB, VEGFA, AKT1, TNF, MMP2, MMP9, STAT3, EGFR, MAPK1, FGF2, CASP3, and others after screening with degree ≥27, betweenness ≥0.08, and closeness ≥0.58 (Figure 2C). Importantly, among the 93 overlapping targets, TGF-β and its receptors (TGF-βR1 and TGF-βR2), as classical profibrotic indicators, were also enriched, which are potential targets of 31 components in the WBT formula (Figure 2D). Collectively, 36 components and their 93 potential targets for PF were predicted.

**TABLE 2:** The 36 components of WBT formula from UHPLC/Q-TOF-MS analysis screened according to PubChem database.

Component ID	Ingredient name	Molecular weight	Molecular formula	Herb source
1	Cryptochlorogenic acid	354.0951	C_16_H_18_O_9_	Zi-wan/Kuan dong-hua
2	Chlorogenic acid	354.0951	C_16_H_18_O_9_	Zi-wan/Kuan dong-hua
3	Amygdalin	457.1584	C_20_H_27_NO_11_	Tao-ren
4	Caffeic acid	180.0423	C_9_H_8_O_4_	Zi-wan
5	Viscidulin I	302.0427	C_15_H_10_O_7_	Huang-qin
6	Calycosin-7-glucoside	446.1213	C_22_H_22_O_10_	Huang-qin
7	Polydatin	390.1315	C_20_H_22_O_8_	Hu-zhang
8	Chrysin 6-C-glucoside 8-C-arabinoside	548.153	C_26_H_28_O_13_	Huang-qin
9	Baicalein 7-O-β-D-glucuronide	464.0955	C_21_H_20_O_12_	Huang-qin
10	Scutellarin	462.0798	C_21_H_18_O_12_	Huang-qin
11	Isochlorogenic acid A	516.1268	C_25_H_24_O_12_	Kuan-dong-hua
12	Isochlorogenic acid B	516.1268	C_25_H_24_O_12_	Kuan-dong-hua
13	5,4′-Dihydroxy-7-methoxyflavone	284.0685	C_16_H_12_O_5_	Huang-qin
14	Isochlorogenic acid C	516.1268	C_25_H_24_O_12_	Kuan-dong-hua
15	Scutellarin methylester	476.0955	C_22_H_20_O_12_	Huang-qin
16	Viscidulin III	346.0689	C_17_H_14_O_8_	Huang-qin
17	Emodin-8-β-D-glucoside	432.1056	C_21_H_20_O_10_	Hu-zhang
18	Baicalin	446.0849	C_21_H_18_O_11_	Huang-qin
19	Salvianolic acid B	718.1534	C_36_H_30_O_16_	Dan-shen
20	Dihydrobaicalin	448.1006	C_21_H_20_O_11_	Huang-qin
21	Norwogonin 7-O-glucuronide	446.0849	C_21_H_18_O_11_	Huang-qin
22	Salvianolic acid A	494.1213	C_26_H_22_O_10_	Dan-shen
23	Chrysin-7-O-β-D-glucuronide	430.09	C_21_H_18_O_10_	Huang-qin
24	Oroxylin A 7-O-β-D-glucuronide	460.1006	C_22_H_20_O_11_	Huang-qin
25	5,7-dihydroxy-6-methoxyflavone 7-O-β-D-glucuronide	460.0955	C_22_H_20_O_11_	Huang-qin
26	Emodin glucoside	432.1056	C_21_H_20_O_10_	Hu-zhang
27	Wogonoside	460.1006	C_22_H_20_O_11_	Hu-zhang
28	Scutellarein	286.0477	C_15_H_10_O_6_	Huang-qin
29	Baicalein	270.0528	C_15_H_10_O_5_	Huang-qin
30	8-methyl-5,7,4′-trihydroxyisoflavone	284.0685	C_16_H_12_O_5_	Huang-qin
31	Astragaloside III	784.4609	C_41_H_68_O_14_	Huang-qi
32	Wogonin	284.0685	C_16_H_12_O_5_	Huang-qin
33	Astragaloside Ⅱ	826.4715	C_43_H_70_O_15_	Huang-qi
34	Oroxylin A	284.0685	C_16_H_12_O_5_	Huang-qin
35	Astragaloside I	868.482	C_45_H_72_O_16_	Huang-qi
36	Emodin	270.0528	C_15_H_10_O_5_	Hu-zhang

**TABLE 3:** The 93 hub targets between 36 active components of WBT formula and pulmonary fibrosis.

No	Gene name	No	Gene name	No	Gene name
1	ABCB1	32	HMOX1	63	PDE4A
2	ACE	33	HRAS	64	PDE5A
3	ADRB2	34	IFNG	65	PLAT
4	AKT1	35	IGF1	66	PLG
5	ALB	36	IGF1R	67	PMS2
6	ANXA5	37	IL2	68	PPARG
7	APEX1	38	JAK2	69	PRSS1
8	ASL	39	KDR	70	PTGS2
9	BMP2	40	KIT	71	PTPN11
10	BMP7	41	KRAS	72	RAF1
11	BPI	42	KRT7	73	RARB
12	BRAF	43	LGALS3	74	REN
13	CALR	44	LTF	75	Rnase3
14	CASP3	45	MAP2K1	76	S100A9
15	CAT	46	MAPK1	77	SCN5A
16	CCL5	47	MAPK14	78	SELE
17	CDC42	48	MAPK8	79	SELP
18	CHIT1	49	MET	80	SERPINA1
19	CHRM3	50	MIF	81	SLC6A4
20	CTSG	51	MMP1	82	SPARC
21	EGFR	52	MMP12	83	SRC
22	ELANE	53	MMP2	84	STAT1
23	F2	54	MMP3	85	STAT3
24	FGF2	55	MMP7	86	TEK
25	FGFR1	56	MMP8	87	TGFB2
26	FGFR2	57	MMP9	88	TGFBR1
27	FHIT	58	MPO	89	TGFBR2
28	GBA	59	MT-CYB	90	TNF
29	GSTM1	60	NOS2	91	TNFSF11
30	GSTP1	61	NOS3	92	TRPV4
31	HDAC2	62	NR1H4	93	VEGFA

### Enrichment Pathways Analysis of WBT Formula

**FIGURE 3:**
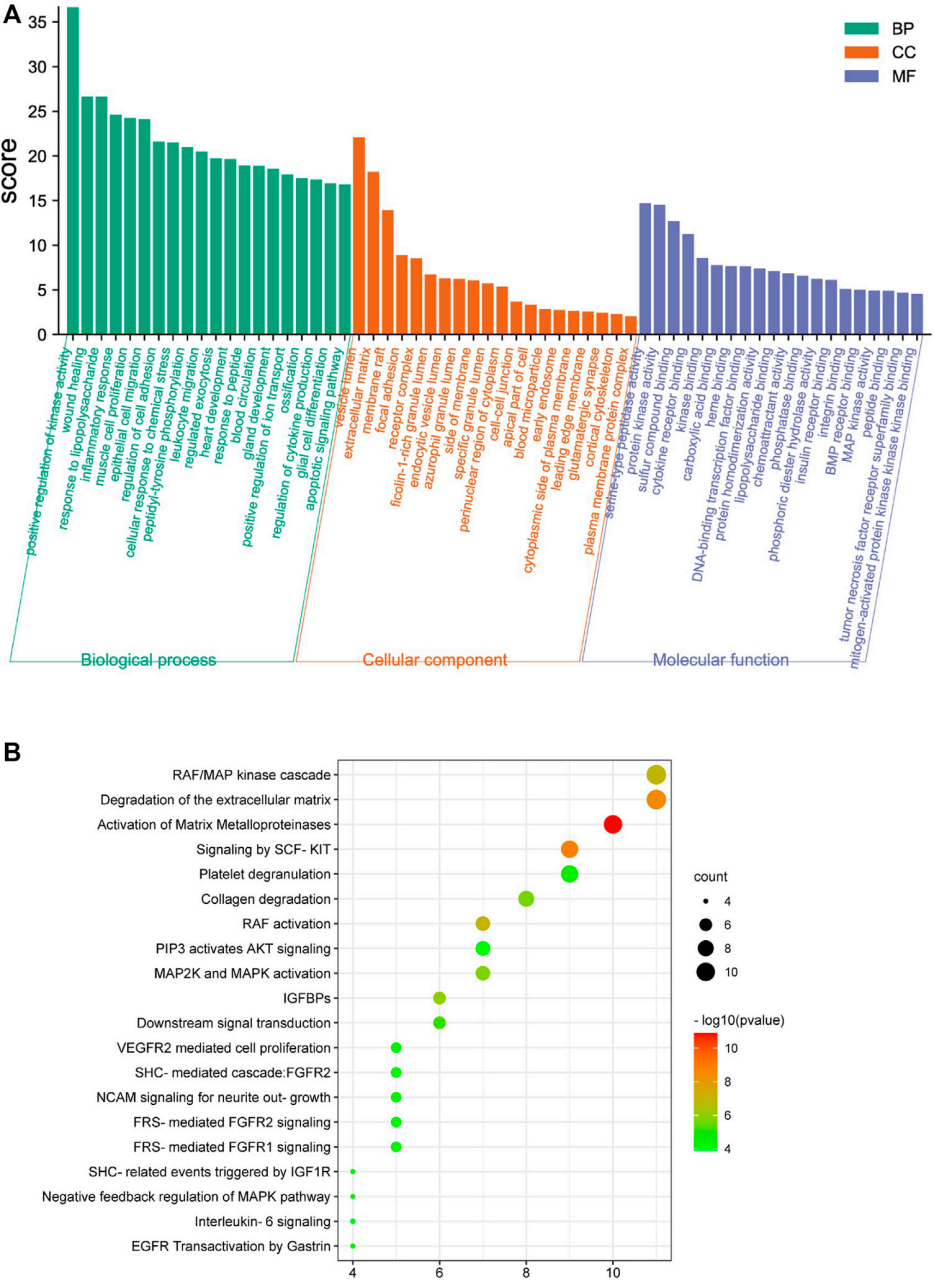
Enrichment analysis of the potential targets of WBT formula against PF was performed by the Metascape and DAVID database. **(A)** The top 20 terms of biological process (BP), cellular component (CC), and molecular function (MF) of WBT formula are shown as a bar diagram. **(B)** The top 20 Reactome pathway terms participating in PF are shown as a bubble diagram by the DAVID database, according to the *p*-value of the calculation.

The Metascape database was used to perform GO enrichment for analyzing the potential mechanisms of the WBT formula. For these mechanisms, the 390 biological processes (BPs), 80 cellular components (CCs), and 105 molecular functions (MFs) were obtained and are shown in Supplementary Tables S5–S7. Moreover, the top 20 entries were selected from BP, CC, and MF, in order of -log *p* value, are shown in Figure 3A. The BPs regulated by WBT included the positive regulation of kinase activity, wound healing, inflammatory response, cytokine production, and the apoptotic signaling pathway. For CCs, CC mainly contained the vesicle lumen, the ECM, and focal adhesion. In the MFs, the targets were mainly involved in peptidase activity, kinase activity, BMP receptor binding, cytokine receptor binding, and other bindings. Furthermore, the DAVID database was used to obtain 91 terms from the Reactome pathway enrichment analysis (Supplementary Table S8). Importantly, the top 20 enrichment pathways significantly involved in the mechanism of WBT formula included the RAF/MAP kinase cascade (KIT, MAPK1, KRAS, TEK, JAK2, HRAS, FGF2, EGFR, IL2, FGFR2, and FGFR1), the degradation of the ECM (MMP1, MMP2, MMP3, MMP7, MMP8, MMP9, MMP12, CASP3, CTSG, PLG, and ELANE), the activation of MMPs (PRSS1, MMP1, MMP2, MMP3, MMP7, MMP8, MMP9, CTSG, PLG, and ELANE), collagen degradation (MMP1, MMP2, MMP3, MMP7, MMP8, MMP9, MMP12, and ELANE), and other pathways (Figure 3B). Remarkably, these potential targets participating in the degradation of ECM were regulated by TGF-β signaling pathway, which is an important regulator in the occurrence and development of fibrosis (Frangogiannis, 2020). Together, the network pharmacology results indicate that WBT may inhibit the TGF-β signaling pathway to regulate ECM production and degradation or other downstream pathways for the blockade of PF progression. However, except for the TGF-β signaling pathway, the mechanisms and targets from the network pharmacology need to be further evaluated.

### WBT Attenuates Pulmonary Fibrosis Progression in BLM-Induced Mice

**FIGURE 4:**
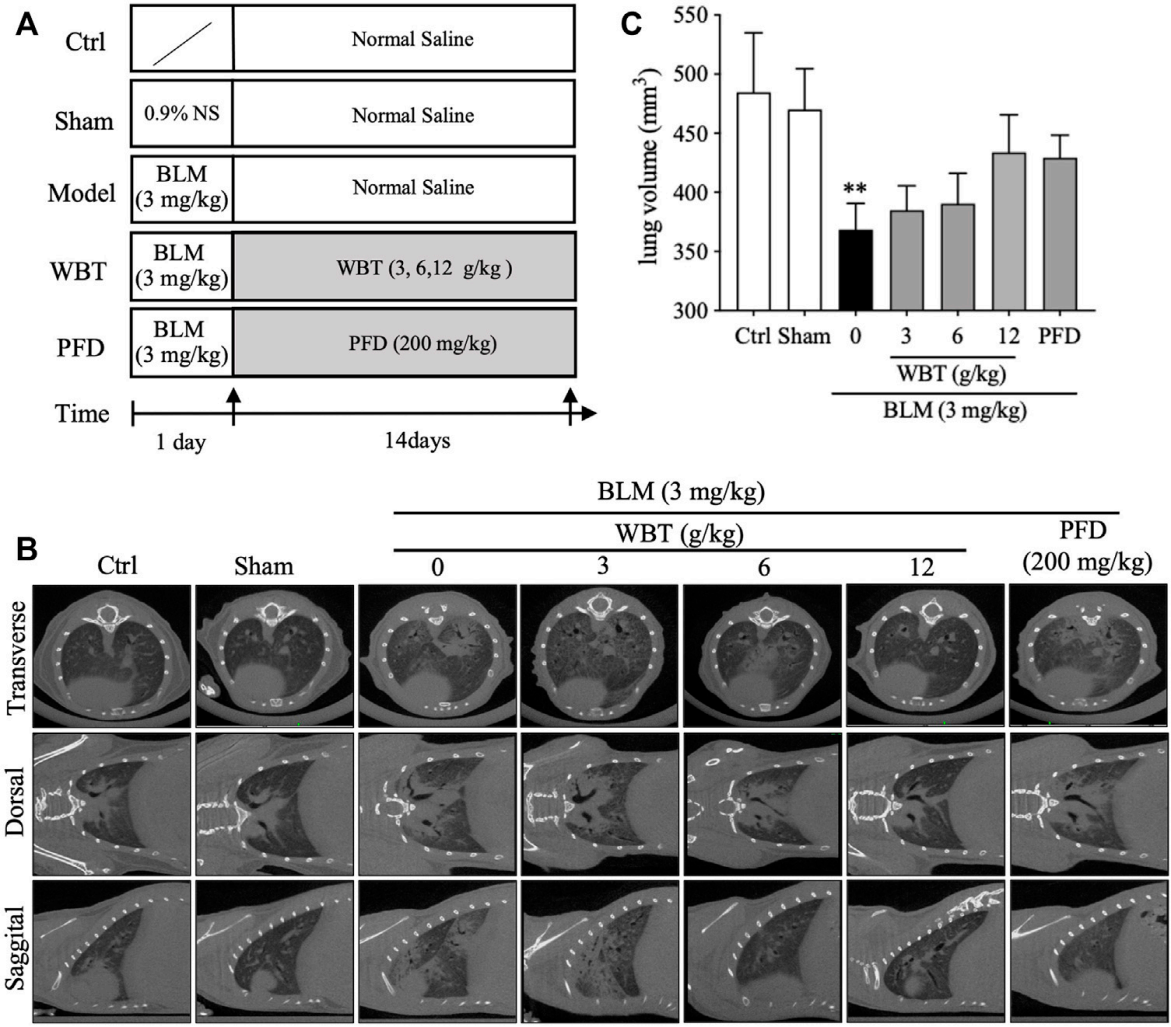
The protective effect of WBT formula on BLM-induced lung injury was evaluated by micro-CT imaging. **(A)** A diagram for the mouse modeling process and WBT or PFD administration is shown. **(B)** After BLM induction and 14-day intervention, the representative *in vivo* micro-CT scans for the lungs from different groups of Ctrl, Sham, BLM, BLM + WBT (3, 6, and 12 g/kg), and BLM + PFD (200 mg/kg) in the transverse, dorsal, or sagittal planes. **(C)** The lung volume of different groups in the BLM-induced mouse model was quantified by the three-dimensional volume rendering technique (n = 4 per group). **p < 0.01, compared to the Sham group. BLM, bleomycin; Ctrl, control; WBT, Wenfei Buqi Tongluo formula; PFD, pirfenidone.

To further assess the protective effect of WBT against PF, mice were treated with WBT or PFD for 14 days after intratracheal injection of BLM (Figure 4A). After the treatment, the lungs of mice in the different groups (Sham, BLM, BLM + WBT, and BLM + PFD) were observed in three different planes by micro-CT scanning. As shown in Figure 4B, the micro-CT imaging shows that lung tissues from blank and sham groups show clear texture, no abnormal density shadow, and a bronchial vascular bundle that runs normally. The interstitial thickening around bronchial vascular bundles in the axial fiber system and the diffuse interlobular septum thickening in the peripheral fiber system can be markedly observed in the both lungs from BLM-induced model group. Moreover, subpleural linear shadows are seen in many places, indicating that BLM-induced lungs have obvious characteristics of ground glass density and grid-like changes. Compared with model group, the lung texture is clearer, and the bronchial vascular bundles are not significantly thickened in the lungs from WBT-treated group. The original diffuse ground glass density and grid-like change in both lungs were obviously inhibited by WBT treatment (Figure 4B). In addition, BLM-induced decreases of lung volume were slightly improved by WBT treatment, compared with that of the model group (Figure 4C). Moreover, the protective effect of WBT in 12 g/kg was significantly better than that in 6 and 3 g/kg. The improvement for the changes of PF by WBT at 12 g/kg was similar with that of PFD at 200 mg/kg. Therefore, in subsequent experiments, the WBT group of 12 g/kg was used for histopathological and IHC analysis.

**FIGURE 5:**
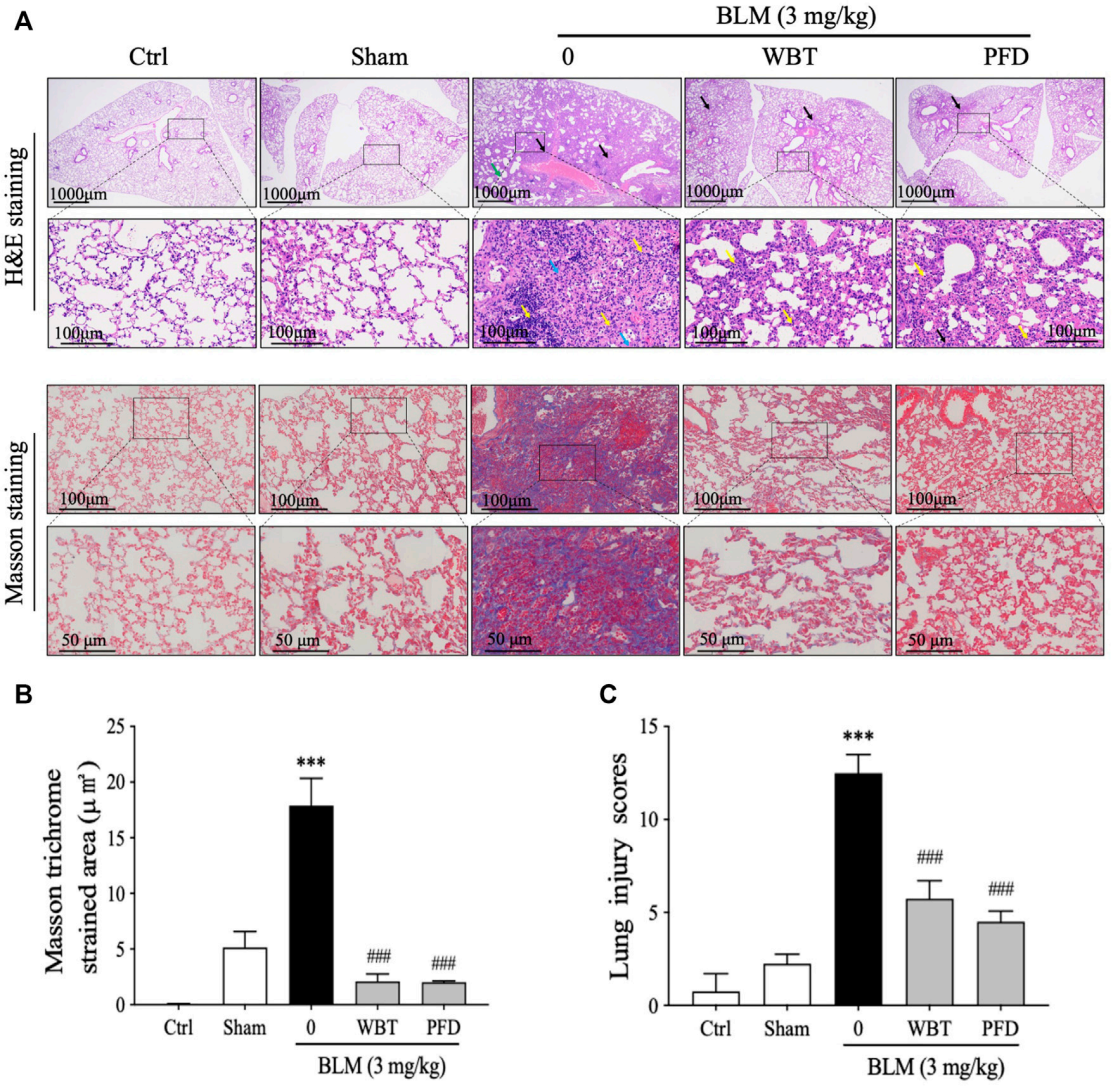
WBT formula attenuated BLM-induced lung fibrosis after 14-day treatment. **(A)** H&E and Masson’s trichrome stainings for the lung tissues from Ctrl, Sham, BLM, BLM + WBT (12 g/kg), and BLM + PFD (200 mg/kg) groups were presented (n = 5). The images in the lower panels (scale bar = 50 μm) of each staining were magnified from the inset of the photomicrographs in the upper panels (scale bar = 200 μm). **(B)** Quantitative analysis of Masson’s trichrome staining in the sections of mouse lungs (n = 5). **(C)** The pathological score of lung injury was analyzed in different groups (n = 5). ***p < 0.001, compared to the sham group; ###*p* < 0.001, compared to the BLM group. BLM, bleomycin; Ctrl, control; WBT, Wenfei Buqi Tongluo formula; PFD, pirfenidone.

H&E and Masson’s trichrome stainings were performed and analyzed to investigate the effect of WBT on histological changes in the lung tissues from BLM-induced mice. The H&E staining results show that many alveolar walls were thickened and accompanied by infiltration of lymphocytes and macrophages in the BLM-induced group, compared with that in the sham group. BLM injection increased the number of inflammatory cells infiltrating around blood vessels and forming vascular sleeves, accompanied by acidic serous substance exudation in the local alveolar cavity (Figure 5A, upper panel). After 14 days of treatment, the extent of tissue destruction was significantly suppressed by WBT or PFD. The bronchial epithelial structure was intact, and the epithelial cells were normal and closely arranged in the WBT group. However, other histological characteristics, including thicker alveolar walls, lymphocyte infiltration, and a small amount of eosinophil and blood in the local alveolar cavity were not obviously improved by WBT (Figure 5A, upper panel). Masson’s trichrome staining confirmed that the blue staining for fibrous collagen deposition as an index of PF in the lung tissues induced by BLM was significantly inhibited by WBT or PFD (Figure 5A, lower panel). The areas of collagen deposition in WBT or PFD-treated lung tissues were markedly lower than that in the BLM-induced group (Figure 5B). Notably, the scores from Mikawar and Ashcroft methods were reduced by WBT on the basis of H&E and Masson’s trichrome stainings, which indicates that WBT can decrease the lung injury induced by BLM (Figure 5C). Together, WBT treatment can improve lung architecture and collagen deposition to block the progression of PF in the BLM-induced mouse model.

### WBT Decreases the Level of TGF-β and Smad3 Phosphorylation in BLM-Induced Mice

**FIGURE 6:**
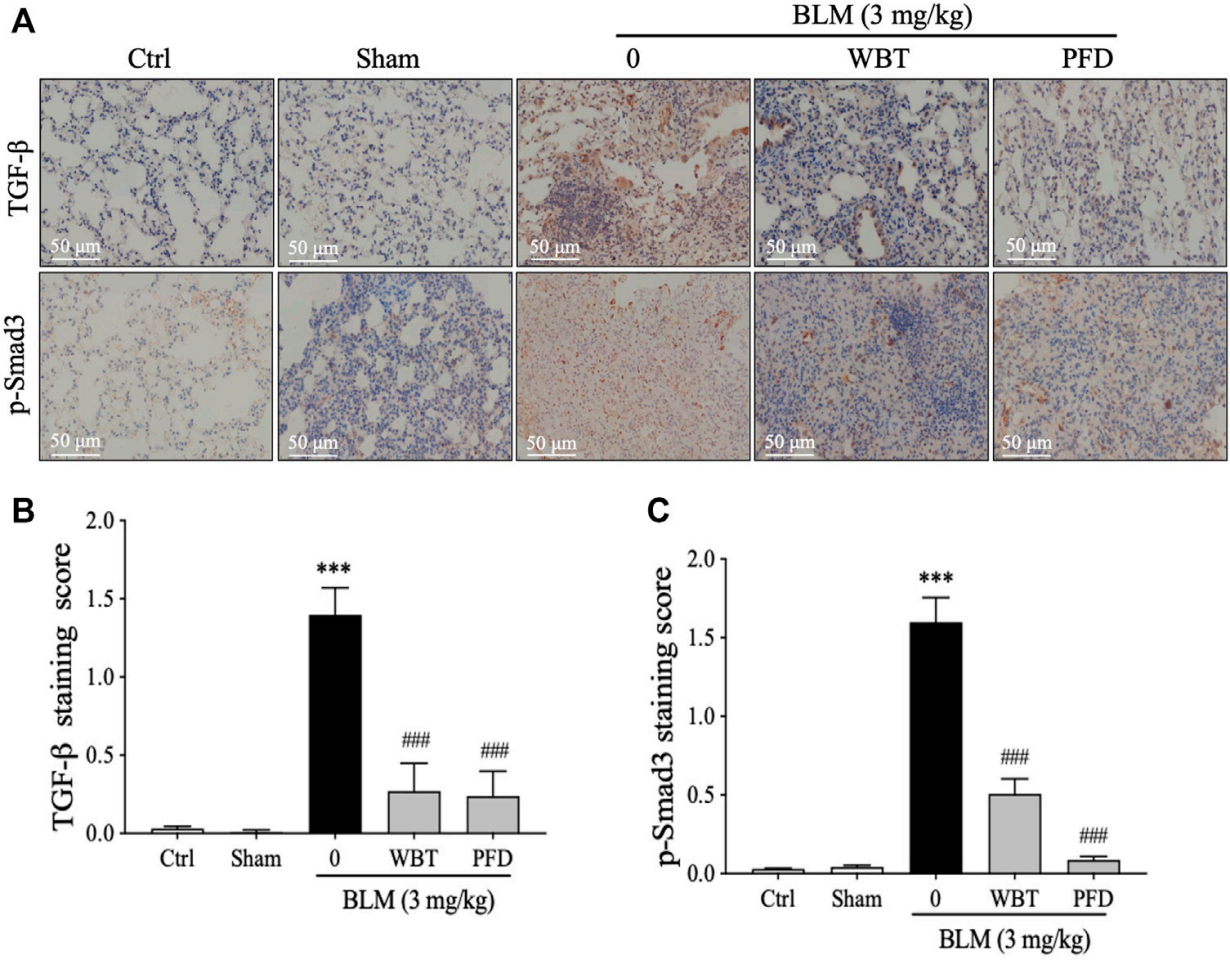
The WBT formula inhibited the TGF-β/Smad3 pathway in the BLM-induced mouse model of lung fibrosis. **(A)** Immunohistochemical assay was used to detect the expression of TGF-β1 and p-Smad3 in the lungs from different groups of control, sham, model, and treatment. Scale bar = 50 μm. **(B,C)** Quantification of relative TGF-β1 and p-Smad3 levels from **(A)** was analyzed (n = 5); ***p < 0.001, compared to the sham group; ###*p* < 0.001, compared to the BLM group. BLM, bleomycin; Ctrl, control; WBT, Wenfei Buqi Tongluo formula; PFD, pirfenidone.

As enriched by the network pharmacology, the TGF-β signaling pathway is critical for the protection of WBT against PF. Therefore, the expressions of TGF-β and p-Smad3 in different lung tissues from the sham, model, or WBT/PFD groups were determined by IHC staining. As shown in Figures 6A,B, the increase of TGF-β level induced by BLM is significantly down-regulated by WBT. Furthermore, the phosphorylation of Smad3 is significantly inhibited by the WBT treatment, compared with the BLM-induced model group (Figure 6A, lower panel, and Figure 6C). In addition, WBT and PFD have similar inhibitory effects in TGF-β and p-Smad3 expressions at the protein level. Collectively, WBT blocks the TGF-β/Smad3 pathway in the lung tissues of BLM-induced mice, which confirms the enrichment of TGF-β pathway for WBT by the network pharmacology.

### WBT Reduces Collagen Accumulation in BLM-Induced Mice and TGF-β1–Induced TC-1 Cells

**FIGURE 7:**
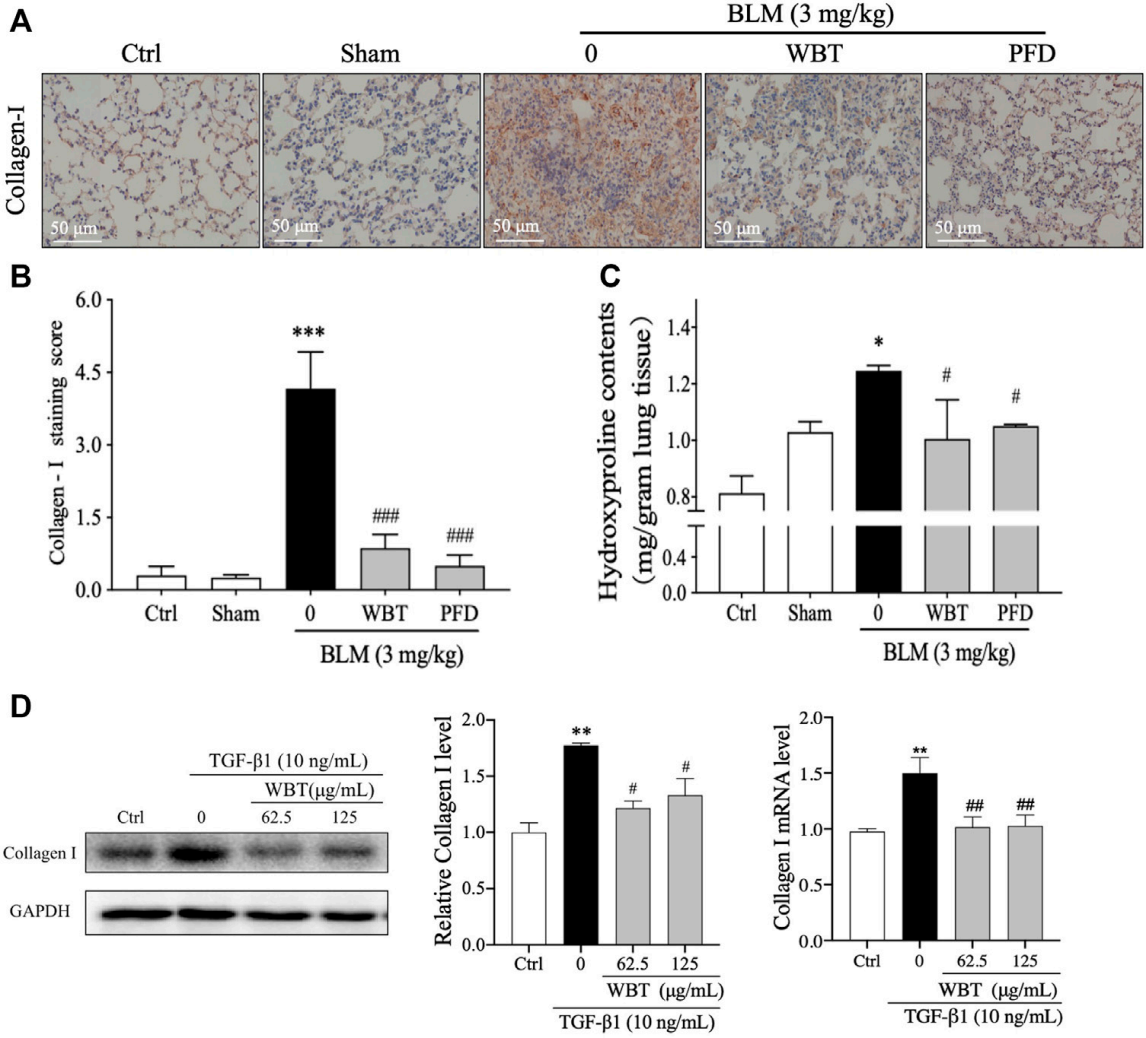
WBT formula inhibited BLM-induced collagen deposition in the mouse and TC-1 cell model of lung fibrosis. **(A)** The level of collagen I was detected by immunohistochemical staining in the mouse lung from different groups. Scale bar = 50 μm. **(B)** The relative collagen I level from **(A)** was quantified (n = 5); ***p < 0.001, compared to the sham group; ###*p* < 0.001, compared to the BLM group. **(C)** The content of hydroxyproline in the lung tissues from different groups was determined by a commercial measurement kit. **(D)** After incubation with TGF-β1 and/or WBT for 48 h, qPCR and Western blot analysis were performed. The relative levels of collagen I in TC-1 cells were calculated and normalized to GAPDH. Data are from three independent experiments. *p < 0.05, **p < 0.01, and ***p < 0.001, compared to the sham group; #*p* < 0.05, ##*p* < 0.05, and ###*p* < 0.001, compared to the BLM or TGF-β1 group. BLM, bleomycin; WBT, Wenfei Buqi Tongluo formula; PFD, pirfenidone (TGF-β1, 10 ng/ml).

Collagen I is an important component of ECM accumulation, an important characteristic of PF (Long et al., 2018). As shown in Figure 3, the collagen degradation pathway is a potential mechanism of WBT against PF, according to the results of network pharmacological analysis. In the BLM-induced mouse model, the level of collagen I was up-regulated by BLM induction, which was significantly decreased by the treatment of WBT or PFD (Figures 7A,B). Moreover, HYP, a special amino acid extracted from collagen, represents the content of total collagen, which is a theoretically guide for the diagnosis and prognosis of PF (Berisio et al., 2004). The measurement of HYP content in mouse lung tissues showed that the HYP level in BLM-induced model group was higher than that in the sham groups, whereas the content of HYP induced by BLM was significantly decreased by WBT treatment (Figure 7C). In addition, in TGF-β1–induced TC-1 cells (the cytotoxicity test of WBT to TC-1 cells is shown in Supplementary Figure S4), the expression of collagen I was also significantly down-regulated by WBT treatment (Figure 7D). Together, these results indicated that WBT can attenuate collagen accumulation *in vivo* and *in vitro.*

### WBT Inhibited EMT in BLM-Induced Mice and TGF-β1–Induced TC-1 Cells

**FIGURE 8:**
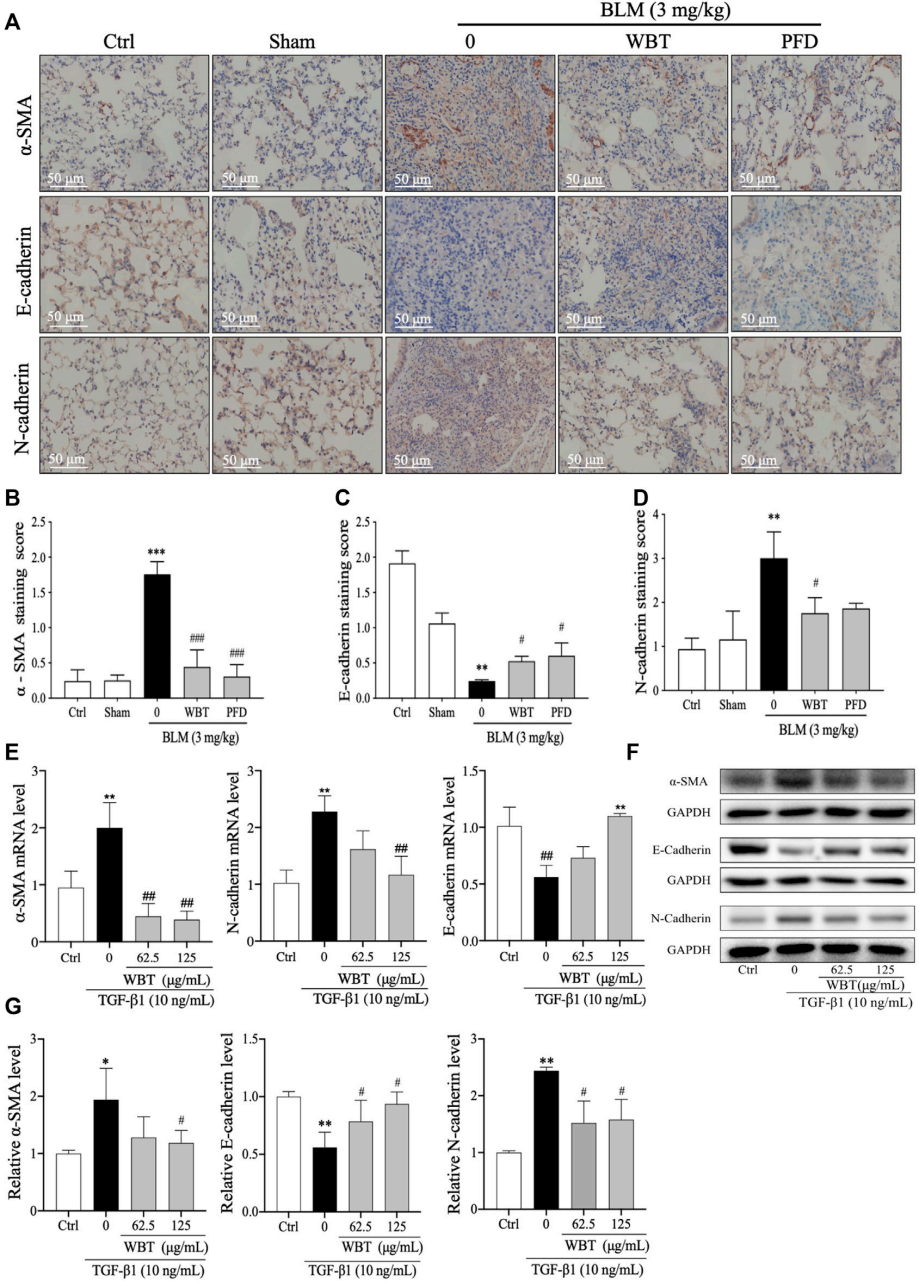
WBT formula inhibited epithelial–mesenchymal transition induced by BLM in the mouse model and TGF-β1 in the TC-1 cell model. **(A)** The levels of α-SMA, E-cadherin, and N-cadherin in the lung tissues from control (Ctrl), sham, BLM, and BLM + WBT/PFD groups were determined by immunohistochemical assay. Scale bar = 50 μm. **(B,D)** The levels of α-SMA, E-cadherin, and N-cadherin from **(A)** were quantified (n = 5). **(E)** After incubation with TGF-β1 and/or WBT for 48 h, qPCR analysis was performed and the relative mRNA levels of α-SMA, E-cadherin, and N-cadherin in TC-1 cells were calculated and normalized to GAPDH. **(F,G)** The protein levels of α-SMA, E-cadherin, and N-cadherin in TC-1 cells treated with TGF-β1 and/or WBT for 48 h were determined by Western blot analysis. GAPDH was used as the loading control. Data are from three independent experiments. *p < 0.05, **p < 0.01, and ***p < 0.001, compared to the sham group; #*p* < 0.05, ##*p* < 0.05, and ###*p* < 0.001, compared to the BLM or TGF-β1 group. BLM, bleomycin; WBT, Wenfei Buqi Tongluo formula; PFD, pirfenidone (TGF-β1, 10 ng/ml).

EMT is a common developmental process in the pathogenesis of PF, which can be induced by TGF-β (Nieto et al., 2016). Consistent with the previous study (Ding et al., 2021), the treatment of WBT significantly inhibited EMT *in vivo* and *in vitro*. In the BLM-induced mouse model, the IHC staining demonstrated that the EMT with the decreased E-cadherin and increased N-cadherin and α-SMA was promoted by BLM, which was blocked by WBT, compared with that of the model group (Figure 8A, middle and lower panels, and Figures 8C,D). In this BLM-induced mouse model, the regulation of WBT on EMT markers was similar to that of PFD (Figure 8). In addition, in TGF-β1–induced TC-1 cells, the changes of α-SMA, E-cadherin, and N-cadherin regulated by the WBT formula at the mRNA and protein levels were similar to those in the BLM-induced mouse model (Figures 8E–G). Together, these data indicate that WBT can inhibit EMT in the BLM-induced mouse model and the TGF-β1–induced cell model.

## Discussion

To explore the chemical components and mechanism of the WBT formula against PF, in this study, we performed UHPLC/Q-TOF-MS analysis, the network pharmacology prediction and experimental validation. According to PubChem database, a total of 36 chemical compounds in WBT powdery extract from UHPLC/Q-TOF-MS analysis were identified or tentatively characterized. Then, the potential targets and mechanisms of these compounds against PF were predicted by the network pharmacology. Furthermore, on the basis of the BLM-induced PF mouse model, the effect of WBT against PF was confirmed by micro-CT imaging and histopathological staining. Importantly, the effects of WBT on the TGF-β1 signaling pathway, EMT inhibition, and ECM degradation were investigated to verify the network pharmacology-based mechanism prediction. Overall, this study was the first to demonstrate that the WBT formula inhibited the TGF-β/Smad3 pathway to reduce EMT and promote ECM degradation against PF.

On the basis of the theories of TCM with tonifying *Qi*, activating blood circulation, dredging collaterals, and clearing heat, the WBT formula was formed and used for the clinical applications of lung-related diseases. Among this formula, *Qi*-tonifying (Huang qi), blood-activating (Dan shen, Dang gui, Chuan xiong, and Di long), and heat-clearing (Huang qin) Chinese medicines are usually used in clinical practice for the treatment of PF, which is consistent with a previous report (Zhang et al., 2018). The anti-PF effect of Huang qi and Dang gui has been confirmed by a systematic review and meta-analysis for randomized controlled trials of IPF (Zhang Y. et al., 2020). In this study, we found that the main chemical components of WBT formula were present in four medicinal herbs: Huang qin, Huang qi, Hu zhang, and Kuan donghua. After UHPLC/Q-TOF-MS analysis and screening by the PubChem database, 36 potential compounds that have been reported as treating PF were obtained, including baicalein, baicalin, scutellarein, scutellarin, salvianolic acid B, polydatin, and chlorogenic acid. As reported previously, baicalein attenuates fibroblast differentiation and collagen production in fibroblasts and rat PF models, which may be mediated by regulating the connective tissue growth factor, miR-21, or the TGF-β/Smad signaling pathway (MacLusky et al., 1986; Gao et al., 2013; Sun et al., 2020). Baicalin ameliorates BLM-induced PF by regulating the PI3K/Akt and TGF-β signaling pathways (Huang et al., 2016; Zhao et al., 2020). The *in vitro* and *in vivo* evidence demonstrates that scutellarein and scutellarin can inhibit PF progression through regulation of the TGF-β/Smad, PI3K/Akt, Bax/Bcl2, or NF-κB/NLRP3 pathways (Miao et al., 2020; Peng et al., 2020). Moreover, four compounds, namely, baicalein, 7-O-β-D-glucuronide, viscidulin I, and viscidulin III, were screened out as the top key compounds by topological property (Supplementary Figure S3) from the network pharmacology, which are from Huang qin (Supplementary Table S1). However, except for baicalein, the effect of other three compounds in anti-PF needs to be further verified by experimentation. Overall, the chemical components of Huang qin may have a potential function in treating PF. In addition, salvianolic acid B from Dan shen, polydatin from Hu zhang, and chlorogenic acid from Zi wan or Kuan donghua were reported to prevent and treat PF by multiple mechanisms, including anti-inflammation, endoplasmic reticulum stress inhibition, and TGF-β/Smad blockade (Hogan and Lee, 1987; Liu et al., 2016; Wang et al., 2017; Jiang et al., 2020). The above findings support the claims that these compounds, identified by UHPLC/Q-TOF-MS analysis, may be active components of WBT for treating PF.

The WBT formula had an obviously inhibitory effect on PF in the cell models (Ding et al., 2021), but the molecular mechanism of WBT remains unclear. According to the results of the PPI network, the potential targets of WBT against PF included ALB, VEGFA, STAT3, MAPK1, MMPs, TGFB2 and its receptors, and FGF2 and its receptors, which participate in many progressions of PF, such as ECM deposition, EMT, and myofibroblast differentiation. Critically, we also found that TGFB2 and its receptors (TGFBR1 and TGFBR2) were potential targets of 31 components identified from WBT. In our study, we evaluated the inhibitory effect of WBT against PF in the BLM-induced mouse model using micro-CT imaging and histopathological analysis and further assess the key roles of TGF-β signaling pathways to explain the molecular mechanism of WBT against PF. The images of micro-CT, H&E, and Masson’s trichrome stainings have proved that WBT had significant inhibition for the lung fibrotic injury after BLM induction. Compared with the BLM-induced model group, the levels of TGF-β and p-Smad3 were significantly inhibited by WBT, which supported the predicted mechanism of WBT from the network pharmacology. For excessive ECM deposition, *in vivo* and *in vitro*, WBT could promote collagen degradation and decrease the content of lung HYP and the expression of collagen I, which could block the progress of PF. The role of WBT on ECM accumulation might be related to the activation of matrix metalloproteinases (MMPs), including collagenases (MMP1 and MMP8), gelatinases (MMP2 and MMP9), and stromelysins (MMP3 and MMP7) (Mahalanobish et al., 2020); however, the specific effect and mechanisms of MMPs need to be further investigated for supporting the prediction of the network pharmacology. Furthermore, some of the chemical components of WBT, such as amygdalin, scutellarin, and salvianolic acid B, have been reported to inhibit the EMT by regulating several markers, including α-SMA, E-cadherin, and N-cadherin (Liu et al., 2016; Wang et al., 2019; Peng et al., 2020). In addition, other signaling pathways, such as FGF, EGFR, VEGFA, and MAPK, play potential roles in the pathogenesis of PF (Iyer et al., 2015; Venkataraman and Frieman, 2017; Koo et al., 2018; Guo et al., 2021), which have been enriched as potential targets of WBT and are involved in cytokine production, cell proliferation, adhesion and migration, ECM degradation, and MMP activation. However, the regulatory effects of WBT on these pathways in anti-PF treatment should be further investigated. More importantly, the direct interaction of those potential compounds from WBT with TGF-β and other targets also needs to be detected in the future.

## Conclusion

In summary, we first time identified 36 chemical compounds of WBT and predicted TGF-β signaling pathway and ECM degradation as potential mechanisms of WBT against PF by the network pharmacology. Furthermore, BLM-induced mouse model and TGF-β1–induced cell model were used to verify the prediction of the network pharmacology and found that WBT treatment can inhibit the levels of TGF-β and Smad3 phosphorylation and subsequently alleviate EMT and ECM accumulation *in vivo* and *in vitro*. These findings indicate that WBT could block the progressive process of PF by inhibiting EMT and prompting EMC degradation *via* TGF-β/Smad3 pathway. This study, for the first time, can provide new insights into the molecular mechanism of WBT for the prevention and treatment of PF in the clinical application.

## Data Availability

The original contributions presented in the study are included in the article/Supplementary Material. Further inquiries can be directed to the corresponding authors.
