# Supplementary material for: Wenfei Buqi Tongluo Formula Against Bleomycin-Induced Pulmonary Fibrosis by Inhibiting TGF-β/Smad3 Pathway
Source: Front Pharmacol. 2022 Jan 21;12:762998. doi: 10.3389/fphar.2021.762998 (PMC8814462; doi:10.3389/fphar.2021.762998)
Supplement: Supplementary file 2 [file Presentation1.pdf]

Wenfei Buqi Tongluo formula prevents bleomycin-induced pulmonary fibrosis by inhibiting TGF-β1/Smad3 pathway

Lu Ding[1*], Yaxin Li[2*], Yingying Yang[3], Siyu Song[2], Hongyu Qi[1], Jing Wang[4], Ziyuan Wang[5], Jiachao Zhao[2], Wei Zhang[6], Linhua Zhao[7], Daqing Zhao[1], Xiangyan Li[1#], Zeyu Wang[6#]

[1]Jilin Ginseng Academy, Key Laboratory of Active Substances and Biological Mechanisms of Ginseng Efficacy, Ministry of Education, Jilin Provincial Key Laboratory of Bio-Macromolecules of Chinese Medicine, Changchun University of Chinese Medicine, Changchun, China. [2]College of Integrated Traditional Chinese and Western Medicine, Changchun University of Chinese Medicine, Changchun, China. [3]Graduate College, Beijing University of Chinese Medicine, Beijing, China. [4]Department of Respiratory, Affiliated Hospital of Changchun University of Chinese Medicine, Changchun, China. [5]College of Traditional Chinese Medicine, Changchun University of Chinese Medicine, Changchun, China. [6]Department of Scientific Research, Changchun University of Chinese Medicine, Changchun, China.[7]Molecular Biology Laboratory, Guang'anmen Hospital, China Academy of Chinese Medical Sciences, Beijing, China.

[*]These authors contributed equally for this article.

[#]Corresponding author: Xiangyan Li, Jilin Ginseng Academy, Key Laboratory of Active Substances and Biological Mechanisms of Ginseng Efficacy, Ministry of Education, Jilin Provincial Key Laboratory of Bio-Macromolecules of Chinese Medicine, Changchun University of Chinese Medicine, No. 1035 Boshuo Road, Changchun, 130017 China. Email: xiangyan_li1981@163.com. Zeyu Wang, Department of Scientific Research, Changchun University of Chinese Medicine, Changchun, No. 1035 Boshuo Road, Changchun, 130017 China. Email: zeyu781022@163.com.

Table S1. Primer sequences for quantitative real-time PCR analysis

**Gene name**	**Primer sequences**
Mouse N-Cadherin-F	AGCGCAGTCTTACCGAAGG
Mouse N-Cadherin-R	TCGCTGCTTTCATACTGAACTTT
Mouse E-Cadherin-F	CAGGTCTCCTCATGGCTTTGC
Mouse E-Cadherin-R	CTTCCGAAAAGAAGGCTGTCC
Mouse α-SMA-F	GTCCCAGACATCAGGGAGTAA
Mouse α-SMA-R	TCGGATACTTCAGCGTCAGGA
Mouse Collagen I-F	GGCAAGAATGGAGATGATGG
Mouse Collagen I-R	ACCATCCAAACCACTGAAGC
Mouse GAPDH-F	AGGTCGGTGTGAACGGATTTG
Mouse GAPDH-R	TGTAGACCATGTAGTTGAGGTCA

Table S2. Retention time (RT), accurate mass, fragment ions, predicted chemical formula, mass error (PPM), source and identification of components in WBT detected by UHPLC/Q-TOF−MS.

NO.	RT (min)	Observed m / z	Adducts	accurate mass	Formula	Mass error (ppm)	MS/MS	Identified compounds	source
1	7.10	474.1622	M-H	475.1690	$C_{20}H_{29}NO_{12}$	2.11	474.1607, 382.0999, 333.9960, 266.0899, 206.0681, 165.0196, 134.0476, 116.9289	Mandelic Amide-β-gentiobioside	Tao-ren
2	7.52	353.0875	M-H	354.0951	$C_{16}H_{18}O_9$	0.57	353.0877, 254.0444, 191.0564, 135.0456	Cryptochlorogenic acid or isomer	Zi-wan/Kuan-dong-hua
3	8.74	353.0875	M-H	354.0951	$C_{16}H_{18}O_9$	0.57	353.0879, 289.0722, 191.0564, 171.0303, 133.0298	Chlorogenic acid	Zi-wan/Kuan-dong-hua
4	9.25	456.1500	M-H	457.1584	$C_{20}H_{27}NO_{11}$	-1.36	456.1513, 323.0984, 221.0667	Amygdalin	Tao-ren
5	9.25	179.0343	M-H	180.0423	$C_9H_8O_4$	-1.12	179.0354, 135.0454	Caffeic acid	Zi-wan
6	9.34	456.1500	M-H	457.1584	$C_{20}H_{27}NO_{12}$	-1.36	456.1513, 323.0984, 221.0667	Mandelic acid-β-gentiobioside	Tao-ren
7	11.72	301.0354	M-H	302.0427	$C_{15}H_{10}O_7$	1.59	271.0249, 255.0300, 243.0299, 211.0423	Viscidulin I	Huang-qin

8*	11.74	491.1186	M+FA	446.1213	$C_{22}H_{22}O_{10}$	-0.83	283.0614, 268.0377, 239.0352, 211.0423	Calycosin-7-glucoside	Huang-qi
9	11.80	389.1240	M-H	390.1315	$C_{20}H_{22}O_{8}$	0.69	389.1245, 376.0099, 227.0715, 185.0610, 143.0506	Polydatin	Hu-zhang
10	11.95	547.1456	M-H	548.1530	$C_{26}H_{28}O_{13}$	0.71	547.1463, 457.1144, 367.0826, 337.0721, 281.0820	Chrysin 6-C-α-L-arabinoside 8-C-β-D-glucoside or isomer	Huang-qin
11	12.20	463.0881	M-H	464.0955	$C_{21}H_{20}O_{12}$	0.78	463.0853, 367.0828, 337.0003, 281.0819, 271.0252	Aicalein B or isomer	Huang-qin
12*	12.32	461.0726	M-H	462.0798	$C_{21}H_{18}O_{12}$	1.34	461.0730, 445.0780, 285.0408, 175.0252	Scutellarin	Huang-qin
13	12.94	547.1454	M-H	548.1530	$C_{26}H_{28}O_{13}$	0.29	547.1455, 529.1348, 457.1138, 427.1032, 367.0823, 337.0717, 309.0766, 281.0816, 267.0661, 251.0712	Chrysin 6-C-β-L-arabinoside 8-C-β-D-glucoside or isomer	Huang-qin
14	13.47	547.1453	M-H	548.1530	$C_{26}H_{28}O_{13}$	0.22	547.1460, 457.1142, 427.1032, 367.0825, 337.0718, 309.0768, 281.0816, 267.0661, 251.0713	Chrysin 6-C-β-D-arabinoside 8-C-β-D-glucoside or isomer	Huang-qin
15	13.73	515.1205	M-H	516.12680	$C_{25}H_{24}O_{12}$	2.91	515.1198, 407.0076, 353.0880, 191.0564, 173.0459, 161.0248, 135.0455	Isochlorogenic acid A or isomer	Kuan- dong-hua

16	14.18	515.1176	M-H	516.12680	$C_{25}H_{24}O_{12}$	-2.72	515.1194, 353.0876, 324.0082, 191.0562, 179.0351, 161.0245, 135.0454	Isochlorogenic acid B or isomer	Kuan- dong-hua
17	15.07	541.1355	M-H	542.14240	$C_{27}H_{26}O_{12}$	1.66	541.1353, 313.0571, 227.07190, 185.0608, 124.0169	Gallic acid polydatin	Hu-zhang
18	16.00	283.0610	M-H	284.06850	$C_{16}H_{12}O_{5}$	1.06	283.0611, 268.0376, 239.0348, 211.0399, 195.0452	5,4'-Dihydroxy-7-methoxyflavone or isomer	Huang-qin
19	16.75	515.1175	M-H	516.12680	$C_{25}H_{24}O_{12}$	-2.91	515.1193, 353.0876, 324.0084, 191.0563, 173.0458, 135.0454	Isochlorogenic acid C or isomer	Kuan- dong-hua
20*	17.61	475.0872	M-H	476.0955	$C_{22}H_{20}O_{12}$	-1.14	475.0883, 412.9950, 381.9777, 339.9676, 284.0327, 255.0300, 227.0357, 183.0450	Scutellarin methylester	Huang-qin
21	18.61	345.0607	M-H	346.0689	$C_{17}H_{14}O_{8}$	-1.04	345.0607, 324.9797, 269.0458	Viscidulin III	Huang-qin
22	18.33	431.0971	M-H	432.1056	$C_{21}H_{20}O_{10}$	-1.72	431.1023, 269.0463, 240.0436, 225.0552, 241.0501	Emodin-8-β-D-glucoside	Hu-zhang
23*	21.45	891.1620	2M-H	446.0849	$C_{21}H_{18}O_{11}$	-0.03	445.0776, 383.0764, 341.0668, 269.0455, 251.0350, 241.0504, 222.0401	Baicalin	Huang-qin
24*	22.28	717.1454	M-H	718.1534	$C_{36}H_{30}O_{16}$	-0.33	717.1452, 519.0933, 339.0510, 321.0405, 293.0455, 279.0300,	Salvianolic acid B	Dan-shen

							265.0505, 249.0556, 221.0607, 185.0246		
25	23.32	447.0945	M-H	448.1006	$C_{21}H_{20}O_{11}$	3.87	447.0928, 383.9940, 271.0613, 243.0663, 225.0559	Dihydrobaicalin	Huang-qin
26	24.25	445.0769	M-H	446.0849	$C_{21}H_{18}O_{11}$	-0.52	445.0776, 381.9781, 340.9755, 324.979, 269.0456, 241.0505, 225.0556, 197.0611	Norwogonin-7-O-β-D-glucuronide or isomer	Huang-qin
27*	24.98	493.1126	M-H	494.1213	$C_{26}H_{22}O_{10}$	-1.76	295.0594, 249.0543, 221.0587, 197.0632, 185.0245	Salvianolic acid A	Dan-shen
28	25.72	429.0834	M-H	430.0900	$C_{21}H_{18}O_{10}$	2.87	429.0831, 403.9677, 308.9851, 253.0507, 209.0607	Chrysin-7-O-β-D-glucuronide	Huang-qin
29	25.99	459.0929	M-H	460.1006	$C_{22}H_{20}O_{11}$	0.22	459.0928, 403.9678, 283.0610, 268.0357, 239.0349, 211.0401	Oroxylin-7-O-β-D-glucoronide	Huang-qin
30	26.47	475.0889	M-H	476.0955	$C_{22}H_{20}O_{12}$	2.44	475.0883, 411.9881, 355.9625, 339.9669, 299.0561, 284.0326, 255.0300, 239.0350, 171.0453, 153.9911	5,2'-Dihydroxy-6-methoxyflavone-7-O-β-D-glucoronide	Huang-qin
31*	27.12	431.0987	M-H	432.1056	$C_{21}H_{20}O_{10}$	2.06	431.0985, 381.9781, 340.9755, 324.9798, 311.0564, 293.0456, 282.0552, 269.0457, 240.0429,	Emodin glucoside	Hu-zhang

							255.0557, 210.0313, 197.0608, 182.0376		
32*	27.75	459.0927	M-H	460.1006	$C_{22}H_{20}O_{11}$	0.87	459.0936, 403.9683, 283.0613, 268.0377, 239.0351, 211.0403, 163.0040	Wogonoside	Huang-qin
33	28.59	489.1039	M-H	490.1111	$C_{23}H_{22}O_{12}$	1.23	489.1039, 313.0718, 298.0484, 283.0250	5,7-Dihydroxy-6,8-methoxyflavone glucoronide or isomer	Huang-qin
34*	30.42	285.0402	M-H	286.0477	$C_{15}H_{10}O_6$	0.98	269.0455, 240.0427, 225.0556, 211.0394, 195.0452, 183.0440	Scutellarein	Huang-qin
35*	31.63	269.0455	M-H	270.0528	$C_{15}H_{10}O_5$	2.01	267.0301, 239.0351, 223.0401, 195.0453	Baicalein	Huang-qin
36	35.50	283.0600	M-H	284.0685	$C_{16}H_{12}O_5$	-2.33	283.0610, 193.0509, 116.9288	5-7-4-Trihydroxyisoflavone	Huang-qin
37*	36.31	829.4567	M+FA	784.4609	$C_{41}H_{68}O_{14}$	-2.28	783.4512, 489.3623, 383.2957	Astragaloside III	Huang-qi
38*	36.59	283.0599	M-H	284.0685	$C_{16}H_{12}O_5$	-2.86	283.0611, 268.0376, 239.0352, 163.0041, 139.0556	Wogonin	Huang-qin
39*	37.02	871.4695	M+FA	826.4715	$C_{43}H_{70}O_{15}$	0.29	818.3521, 481.0925, 352.0584	Astragaloside II	Huang-qi
40*	37.24	283.0598	M-H	284.0685	$C_{16}H_{12}O_5$	-3.07	283.0610, 268.0369, 239.0348, 211.0404	OroxylinA	Huang-qin

41*	38.26	913.4809	M+FA	868.482	$C_{45}H_{72}O_{16}$	1.27	913.4770, 879.3804, 837.3740, 819.3592, 311.2230, 269.0454, 170.0042, 116.9287	Astragaloside I	Huang-qi
42*	38.91	269.0452	M-H	270.0528	$C_{15}H_{10}O_5$	0.71	269.0455, 240.0429, 255.0557, 211.0400, 195.0454, 182.0374, 171.0451, 157.0660, 116.9290	Emodin	Hu-zhang

* *The identification was certified by reference standards.*

Table S3. The contents of six key compouns in WBT extract were measured by HPLC analysis.

Component ID	Compound name	Compound content (mg/g)	WBT (12g/kg)	Herb source
1	Calycosin-7-glucoside	0.4499	5.3988	Huang-qin
2	Baicalin	17.2408	206.8902	Huang-qin
3	Salvianolic acid B	3.3389	40.0668	Dan-shen
4	Salvianolic acid A	0.9600	11.5200	Dan-shen
5	Emodin-8-β-D-glucoside	1.0735	12.8820	Hu-zhang
6	Wogonoside	6.7344	80.8128	Hu-zhang

Table S4-S8. Detailed results of network pharmacological analysis. (Uploaded as a separate Excel file).

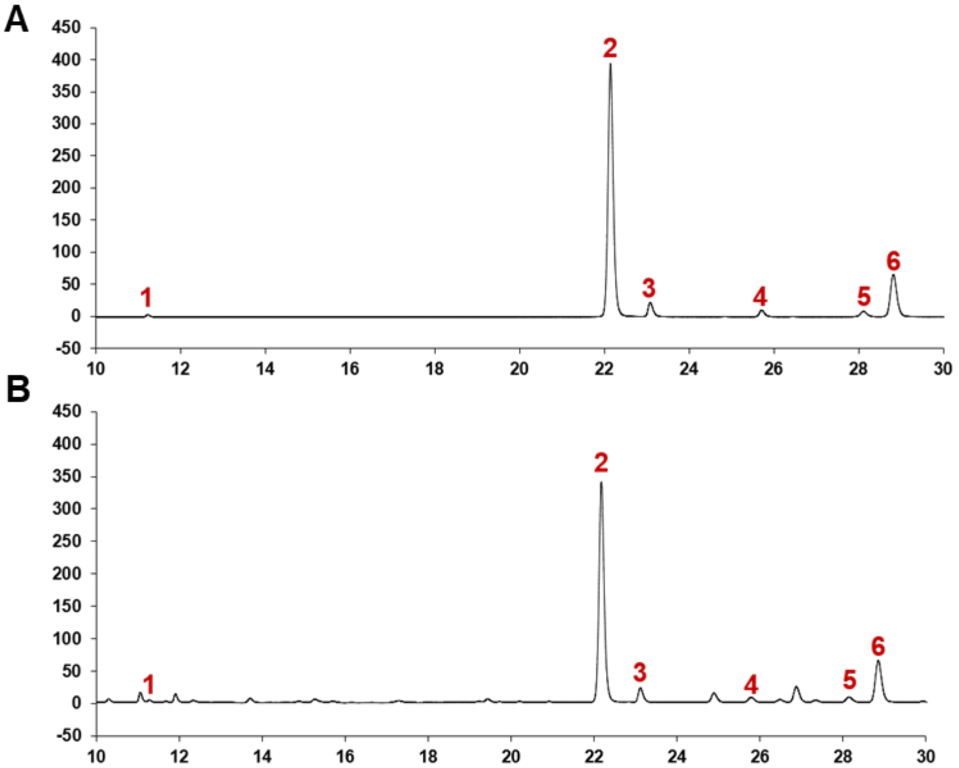

Figure S1. The quantitative analysis of six key compounds in WBT extract. **(A)** The chromatogram of standard mixture. **(B)** The chromatogram of the WBT extract. 1: Calycosin-7-glucoside, 2: Baicalin, 3: Salvianolic acid B, 4: Salvianolic acid A, 5: Emodin-8-β-D-glucoside, 6: Wogonoside.

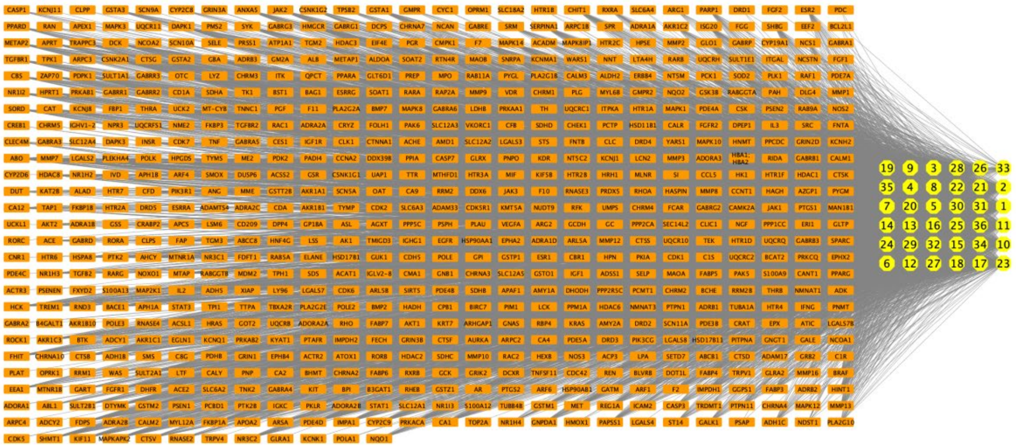

Figure S2. Construction of the network of 36 components–687 potential targets. The yellow octagons represent the IDs of 36 components, and the orange rectangles represent potential therapeutic targets of WBT.

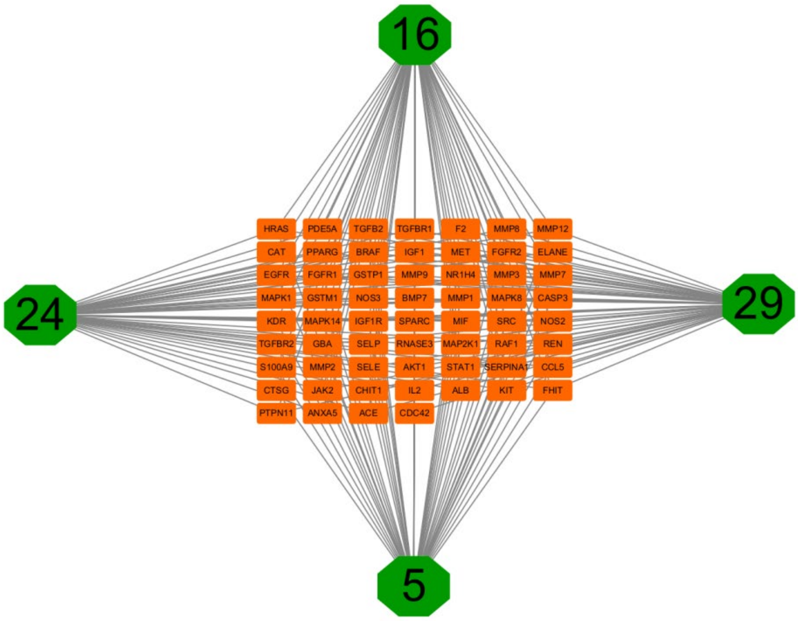

Figure S3. Construction of the network of top four components–overlapping targets (the degree ≥ 48 of components was selected as top four). The green hexagons represent IDs of components, and the orange rectangles represent potential therapeutic targets of WBT.

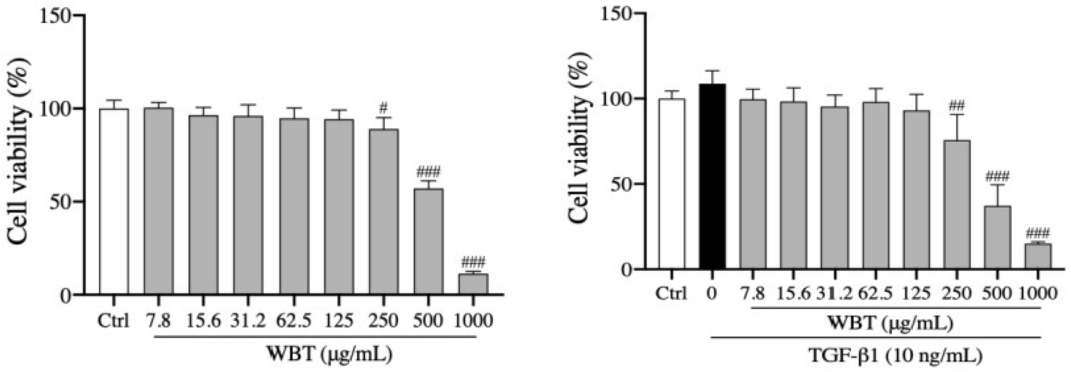

Figure S4. WBT inhibited the proliferation of TGF-β1-stimulated TC-1 cells. **(A)** After treatment with different concentrations of WBT for 48 h, the cytotoxicity of WBT in TC-1 cells was evaluated by MTT assay. **(B)** In TGF-β1-induced-TC-1 cells, the effect of different concentrations of WBT on cell viability after treatment for 48 h was evaluated by MTT assay. Data are shown as mean ± standard deviation from three independent experiments. #*P* < 0.05, ##*P* < 0.01 and ###*P* < 0.001 versus the Ctrl or TGF-β1-induced model group.
